# Simulation in Neurology Residency: Tools to Succeed but Still Mountains to Overcome

**DOI:** 10.1212/NE9.0000000000200167

**Published:** 2024-11-14

**Authors:** Daniel S. Harrison, Nikita Chhabra

**Affiliations:** From the Department of Neurology (D.S.H.), Boston University Medical Center, MA; and Department of Vascular Neurology (N.C.), University of Michigan, Ann Arbor.

Over the past 5 years, there has been an increase in the number of publications evaluating and discussing simulation in clinical neuroscience. These generally fit into 1 of 3 categories: those evaluating the success of a simulation course to teach a neurologic topic at a single institution (i.e., simulation as a formative experience), those evaluating the use of simulation as an assessment tool (i.e., simulation as a summative experience), and those summarizing existing simulation literature and suggesting future directions based on expert opinion.^1-3^ These latter works review the current state of simulation in neurology without the benefit of considering interventions that did not pass peer review, were conducted but never published, or were never even developed with intention to disseminate. As such, while the expert-recommended future directions included therein adequately take into account the research landscape of simulation in clinical neuroscience, they are based on a partially obscured view of all simulation-based neurologic education in practice.

In this issue of *Neurology*® *Education*, Ghoshal et al.^4^ report a description of the current use of simulation-based medical education (SBME) in neurology residency and an assessment of perceived barriers to expanding SBME. The authors surveyed adult neurology residency program directors (PDs), appropriately citing the 44% response rate as a risk of bias toward programs engaging in SBME. However, this rate is almost double that of a previously published investigator-initiated PD survey in neurology.^5^ The authors should be commended on what is likely the most comprehensive description of the landscape of simulation in neurology to date. Most participants reported using SBME in their residencies and creating their own cases, focused on topics in vascular and emergency neurology. Common perceived barriers included limited faculty protected time, resident availability, financial support, and faculty training in simulation. The authors suggest integrating simulation into departmental budgets for quality and safety, collaboration among institutions using SBME, and development of a neurology case library as strategies to address these barriers.

Nearly all respondents reported that a neurology simulation case repository would benefit their program. This has the potential to be a valuable resource to directly address the barriers of limited faculty time and lack of training in development of simulation curricula. However, there are unanswered questions that should be addressed. Will a peer-review process be implemented to ensure quality of cases? If so, this would also allow case developers to claim credit for their work, which would be valuable for academic promotion, especially for clinician-educators. How will this improve on existing peer-reviewed libraries including neurology simulation cases, such as MedEdPORTAL? Will previously peer-reviewed neurology cases from these libraries be eligible for inclusion? Will cases require standard formatting with detailed protocols specifying precisely how each scenario should be run? Paradoxically, this could increase the amount of time spent by some faculty members on simulation work. Perhaps most importantly, will a “shared simulation” that is designed and debriefed by an expert have the same impact on learner outcomes when delivered by an educator without formal simulation training at another institution? Education is context dependent. The gaps of one learner group may ultimately differ from another, and the ability for a standardized case to meet the needs of learners that is context dependent is unclear. These questions should be carefully considered as this resource is developed.

The authors discuss creation of a simulation consortium as another possible facilitator of simulation implementation, highlighting the success of the International Network for Simulation-based Pediatric Innovation, Research, & Education.^6^ This could enable high-quality collaborative research between neurology educators with an eventual downstream effect of increasing funding for multicenter simulation projects. The work by Ghoshal et al. highlights potential research targets for such a consortium, including development and evaluation of SBME as a component of health equity training, which has been previously suggested.^1^ However, this sort of research collaboration will not have immediate effects on faculty protected time, resident availability, financial support, or faculty training in simulation. In the meantime, the consortium should also provide mentorship opportunities for junior faculty members and trainees facing barriers to implementation of SBME at their institutions. A “Neurology in Simulation” interest group already exists through the Society for Simulation in Healthcare, but this forum may not be accessible to those who are not already established simulation educators. A simulation consortium within the American Academy of Neurology would be more pragmatic and should be considered.

There are additional possible facilitators of SBME, which the authors do not discuss. Artificial intelligence (AI), including the use of large language models (LLMs) to develop virtual patients (VPs), has been explored.^7^ Although available LLM-generated VPs are of lower quality than SBME designed by experts, they are cheap, require limited faculty time, could reach a global audience, and represent an important step toward high-fidelity, AI-enhanced simulation. A more established educational intervention, near-peer teaching, is also not discussed by the authors. Implementation of near-peer–led simulation similarly has the potential to both alleviate faculty teaching time and create a pipeline of graduating residents with training in SBME.^8^

Now that we know which mountains must be overcome, we should develop the tools to succeed. We need an evidence-based case library and a globally accessible simulation consortium. A combination of established tools (such as near-peer educators) and novel interventions (such as incorporation of AI) is also needed to effectively expand simulation in the clinical neurosciences. SBME has the potential to improve learner knowledge, confidence, and competence in the management of neurologic diseases.^2^ We owe it to our learners to ensure that they have access to these high-impact interventions.
